# The Relationship Between Psychological Contract Breach and Employees’ Counterproductive Work Behaviors: The Mediating Effect of Organizational Cynicism and Work Alienation

**DOI:** 10.3389/fpsyg.2018.01273

**Published:** 2018-07-27

**Authors:** Shuang Li, Yang Chen

**Affiliations:** School of Management, China University of Mining and Technology, Xuzhou, China

**Keywords:** psychological contract breach, organizational cynicism, work alienation, counterproductive work behavior, mediating effect

## Abstract

Psychological contract breach (PCB) may trigger negative attitudes in employees and ultimately cause further negative behaviors. By drawing on social exchange theory, this study aims to explore the link between PCB and counterproductive work behavior (CWB) by focusing on the mediating role of organizational cynicism and work alienation. We administered a cross-sectional survey of 484 energy company front-line employees. The conceptual model was examined via structural equation modeling. The results suggested that organizational cynicism and work alienation sequentially mediated the relationship between PCB and CWB. This study elucidated the mechanisms underlying the relationship between PCB and CWB by introducing negative attitudes (i.e., organizational cynicism and work alienation) into the model, and offered further evidence that organizations should pay more attention to employees’ PCB and negative attitudes in order to reduce their CWB.

## IntroductIon

In the past few decades, researchers have examined counterproductive work behaviors (CWBs). These behaviors have been labeled differently in different studies, such as organizational misbehavior (Vardi and Wiener, 1996), workplace deviance (Robinson and Bennett, 1997), workplace aggression (Neuman and Baron, 2005), and antisocial behavior (Robinson and O’Leary-Kelly, 1998). All of these behaviors were defined differently, but they share the common theme of being voluntary acts harming or intending to harm organizations and their members (Giacalone and Greenberg, 1997; Spector and Fox, 2005). Thus, all of these definitions fall into the category of CWBs (Sulea et al., 2015). These can be defined as behaviors that voluntarily breach the norms of an organization, and contradict the legitimate interests of an organization or its members (Sackett, 2002). According to Robinson and Bennett (1995), CWBs can be divided into two types according to their target: One is where the CWB is aimed at individual members of the organization (CWB-I); the other is where the CWB is aimed at the organization itself (CWB-O). The former is interpersonally-oriented and may involve bad behaviors toward co-workers, e.g., aggression, offending someone, being impolite and withholding crucial information. The latter is oriented toward the organization and involves behaviors such as theft, sabotage, absenteeism, and safety procedure violations (Bennett and Robinson, 2000; Spector, 2005; Hystad et al., 2014; Cohen, 2016).

Counterproductive work behavior is a key cause of inefficiency and can cause enormous financial losses to enterprises (Tian et al., 2014). For example, in the United States, 33–75% of employees engage in different kinds of CWB (Harper, 1990), which cause losses of 1 trillion dollars each year. These behaviors include theft (120 billion dollars), workplace violence (4.2 billion dollars), and fraudulent activities (more than 900 billion dollars; Banks et al., 2012). Unsurprisingly considering these high costs, researchers have performed many studies on CWBs, exploring CWBs from numerous perspectives. For example, from the standpoint of equity and justice theories, the study of Aquino et al. (1999) confirmed that interactional justice had a negative effect on CWB-O and that distributive and interactional justice had effects on CWB-I. In addition, examining the health impairment process in relation to the Job Demand-Resources (JD-R) model framework, Ceschi et al. (2016) found that the relationship between job demands and CWB was not only mediated by burnout but also moderated by personality traits (i.e., grit and honesty-humility). From a social exchange perspective, Colbert et al. (2004) found that the perception of a supportive work environment was negatively related to CWB. However, few empirical studies have been done from the perspective of the employment relationship. As a framework for understanding the employment relationship, the concept of the psychological contract has gained more attention (Conway and Briner, 2009; Cassar and Briner, 2011). The psychological contract has been defined as the terms and conditions of the reciprocal exchange relationship between an employee and organization, and mutual expectations held by them (Kotter, 1973; Rousseau and Tijoriwala, 1998). If one party perceives that another party has failed to fulfill its obligations or promises, then psychological contract breach (PCB) takes place (Robinson and Rousseau, 1994; Morrison and Robinson, 1997). Given that the employer retains more power (e.g., decision-making) than common employees, he or she can set rules to force employees to fulfill their obligations or promises; hence, the employer hardly perceives PCB. Therefore, we only pay attention to the effects of PCB perceived by employees. With organizational changes such as delayering, downsizing and redundancy, employees may more easily perceive PCB than ever before (Sturges et al., 2005).

We defined employee perception of PCB as an employee perceiving that his or her organization has failed to fulfill its obligations or promises (Rousseau, 1989). Prior studies have suggested that PCB has an effect on work-related attitudes (Bal et al., 2008) and behaviors (Topa Cantisano et al., 2008; Quratulain et al., 2016), such as diminished job satisfaction (Wang and Hsieh, 2014), citizenship behavior (Hejazi, 2016), organizational commitment (Schmidt, 2016), and augmented turnover intentions (Raja et al., 2004). Moreover, previous studies have mainly investigated the role of positive attitudes (e.g., trust, job satisfaction, organization commitment, etc.) that have played a role in the link between PCB and work-related behaviors. A small number of studies have suggested that PCB is positively related to CWB. More specifically, PCB can trigger discretionary absenteeism (Deery et al., 2006), anti-citizenship behavior (Kickul, 2001) and a decrease in in-role job performance (Turnley and Feldman, 2000). Nevertheless, the relationship between PCB and CWB has not been well-examined (Zhao et al., 2007; Jensen et al., 2010). To our knowledge, within this small body of research, only a limited range of CWBs have been examined, with a lack of consideration of the role of negative attitudes on engagement in CWB. To fill this gap, in the present study we attempted to reveal the mechanisms for the relationship between PCB and CWB by taking negative attitudes as mediators.

Social exchange theory states that the parties in an exchange relationship provide benefits to one another in the form of tangible or intangible benefits (Blau, 1964). This exchange relationship follows the norm of reciprocity. The reciprocal norm means that when an individual gets favorable treatment by one party, then it is required of him or her to offer favorable treatment in return (Gouldner, 1960). This also applies if one party receives unfavorable treatment from the other. That is to say, when an individual perceives unfavorable treatment, the individual may reciprocate with negative treatment or poor behavior (Eisenberger et al., 2004; Huang et al., 2016).

Based on social exchange theory, we know that employees with PCB may believe that they are unfavorably treated by their organization, which could elicit negative attitudes and then lead to negative behaviors. Moreover, when PCB comes into being, employees may develop a belief that the organization lacks integrity (Johnson and O’Leary-Kelly, 2003; Zhao et al., 2007) and lose interest in their work (Spindler, 1994). Therefore, we use negative attitudes, organizational cynicism, and work alienation as mediators to reveal the mechanisms of the PCB-CWB relationship.

## Literature Review and Hypotheses

### PCB and CWB

Based on social exchange theory (Blau, 1964), employees may respond to PCB with negative job attitudes, and these would make employees more prone to engaging in CWBs (Law and Zhou, 2014), such as absenteeism (Daouk-Öyry et al., 2014), withdrawal behaviors (Hanisch and Hulin, 1990), and deviant work behavior (Bordia et al., 2008). Moreover, previous research has confirmed that PCB can trigger discretionary absenteeism (Deery et al., 2006) and anti-citizenship behavior (Kickul, 2001). Based on the analysis of 300 doctors and nurses in Pakistan, Ahmed et al. (2013) found that PCB had a significantly positive direct effect on doctors’ and nurses’ CWBs. When perceiving PCB, employees may become angry and frustrated (Eckerd et al., 2013). These negative emotions can elicit employees’ CWB (Fox and Spector, 2002; Penney and Spector, 2005). Moreover, PCB means that employees perceive a discrepancy between what they were promised by the organization and what they have received. Thus, to remove the imbalance, they attempt to reduce effort. In the end, this leads to CWB (Mount et al., 2006; Jensen and Ryan, 2010). Therefore, we proposed that:

**Hypothesis 1:** PCB will be positively related to CWB.

### The Mediating Role of Organizational Cynicism

Organizational cynicism can be described as a negative attitude of employees to their employing organization, which comprises three dimensions: (1) a belief that the organization lacks integrity; (2) negative affect toward the organization; (3) tendencies to perform disparaging and critical behaviors toward the organization (Dean et al., 1998). Drawing on the definitions of Andersson (1996), Dean et al. (1998), and Johnson and O’Leary-Kelly (2003) claimed that organizational cynicism exists as a result of organizational members’ perception of their organization lacking integrity.

Existing research suggests that PCB is an important predictor of organizational cynicism (Andersson, 1996; Dean et al., 1998; Chrobot-Mason, 2003). For instance, in a study of 279 employees, Bashir and Nasir (2013) found a positive relationship between these variables. When PCB occurs, employees perceive that one or more responsibilities of the organization are not being fulfilled (Morrison and Robinson, 1997). When an organization does not keep its promises or obligations, employees may question its integrity (Robinson and Rousseau, 1994), then organizational cynicism occurs (Andersson and Bateman, 1997). Therefore, we hypothesized the following:

**Hypothesis 2:** PCB will have a positive effect on organizational cynicism.

Studies have demonstrated significant positive relationships between organizational cynicism and CWB (James et al., 2011; Ahmed et al., 2013). James (2005) found that organizational cynicism exerted a positive effect on CWBs of teachers. Moreover, as per the view of Bashir (2011) that negative behavior follows negative attitudes, organizational cynicism is positively related to CWB (Ewis, 2014). In addition, as per social exchange theory, employees with a high level of organizational cynicism are frustrated, believing that their organization is exploitive and self-centered and think they get unfavorable treatment. As feedback, the employees who show organizational cynicism tend to slander the organization (Wilkerson et al., 2008).

**Hypothesis 3:** Organizational cynicism will be positively related to CWB.

Ajzen (1989) points out that attitudes serve as a bridge between perception and behavior. What is more, social exchange theory suggests that when individuals perceive that they get treated unfavorably by another party, they will reciprocate with negative attitudes and behaviors. In the present study, taking Hypothesis 1–3 into consideration, when employees perceive PCB they will judge they were treated unfavorably by the organization, which causes them to believe that the organization lacks integrity. In such cases, employees will show organizational cynicism and then engage in CWB (Jiang et al., 2017a). In addition, Steele’s (2014) study confirmed that organizational cynicism mediated the relationship between PCB and workplace behaviors. This led us to suggest the following hypothesis:

**Hypothesis 4**: Organizational cynicism will mediate the link between PCB and CWB.

### Work Alienation as a Mediator

The origins of work alienation can be traced back to Marx (1844). Marx held the view that workers do not care about the work they do, as they do not control the process. Because of this, work alienation occurs (Seybolt and Gruenfeld, 1976). In this particular study, we describe work alienation as a response to organizational conditions (Zeffane, 1993) and the psychological state that comes about if employees are estranged from their work role (Ashforth, 1989; Hirschfeld and Feild, 2000; Nair and Vohra, 2009). In general, work alienation is caused by needs, values, ideals, desires, or expectations not being satisfied (Mottaz, 1981).

Based on previous studies (e.g., Vickers and Parris, 2007; Archibald, 2009), we assumed that PCB would cause work alienation, as suggested by Cullinane and Dundon (2006). This is because, if PCB occurs, employees take the view that work does not contribute to the realization of personal needs or goals (Hirschfeld and Feild, 2000; Saks, 2006), thus causing work alienation (Mottaz, 1981). Moreover, research has found that the greater the PCB, the less meaningful work is perceived as, resulting in higher work alienation (Nair et al., 2010). This suggests the following hypothesis:

**Hypothesis 5:** PCB will be positively related to work alienation.

Conservation of resources (COR) theory points out that all individuals tend to gain and keep the resources they cherish. When these resources are threatened, lost or insufficiently compensated (Hobfoll, 1989), then stress reactions occur. To prevent more of their resources from depleting, individuals may respond to stress by using the remaining resources to gain new and more resources, or withdrawing their efforts (Halbesleben and Bowler, 2007; Ng and Feldman, 2012; Kiazad et al., 2014). In accordance with this point, employees with work alienation think they cannot obtain the resources they value (e.g., job control, support, and esteem; Kanungo, 1978). To restore loss or obtain new resources, employees will reduce effort, which causes sabotage or absenteeism (Kanten and Ulker, 2013; Amazue et al., 2014).

In research with 1,117 participants, Yang et al. (2001) found that work alienation could lead to drinking behaviors in the workplace. Cummings and Manring (1977) found that “powerlessness” and “meaninglessness” (two dimensions of work alienation) were positively related to both absence and tardiness. In addition, work alienation can lead to decreased conscientiousness (Kanungo, 1981; Sulu et al., 2010), and low levels of conscientiousness can predict employees’ CWBs, such as absenteeism and dishonesty (Salgado et al., 2013). Taking all this into consideration, we proposed the following hypothesis:

**Hypothesis 6**: Work alienation will be positively related to CWB.

Employees who perceive PCB may think that an organization has failed to keep a promise, and their expectations are unmet. According to the reciprocity norm, when perceiving unfavorable treatment (e.g., unmet expectations), individuals may reciprocate with negative treatment or poor behavior (Eisenberger et al., 2004; Huang et al., 2016). Taking the reciprocity norm and our hypotheses (H1, H5, and H6) into consideration, employees with high levels of PCB should be more prone to exhibiting work alienation and engaging in CWB. Therefore, we proposed the following hypothesis:

**Hypothesis 7**: Work alienation will mediate the relationship between PCB and CWB.

Organizational cynicism may have a direct link to work alienation (Dean et al., 1998). Some studies have confirmed that organizational cynicism brings work alienation with it (e.g., Koçoğlu, 2014; Jiang et al., 2017b). Organizational cynicism has a positive effect on work alienation, and as the level of organizational cynicism increases, the level of work alienation increases as well (Abraham, 2000; Turan, 2011; Kasalak and Bilgin Aksu, 2014). Once employees form cynicism toward an organization, they may show work alienation in response to this cynicism (Maslach and Schaufelli, 1993). For example, Jiang et al. (2017b) found that front-line production workers with high organizational cynicism are prone to showing work alienation. Yıldız and Şaylıkay (2014) came to the same conclusion in their study of bank employees. Accordingly, we hypothesized:

**Hypothesis 8**: Organizational cynicism will be positively related to work alienation.

**FIGURE 1:**
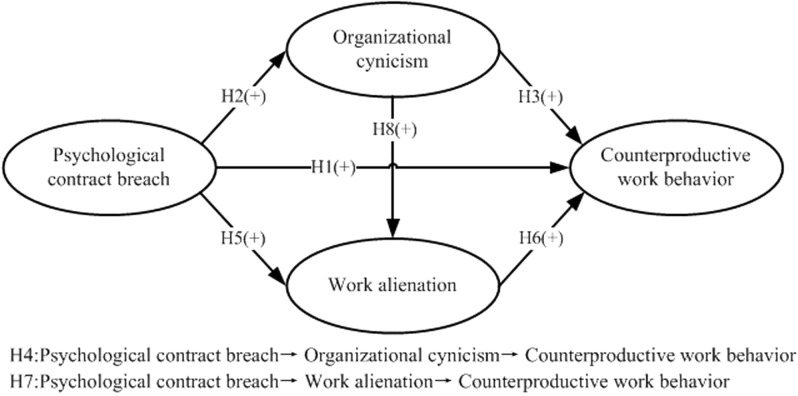
Hypothetical model.

Based on the social exchange theory (Blau, 1964) and previous studies, we developed a multivariate model to examine our hypotheses, specifically the proposed mediating effects of organizational cynicism and work alienation in the relationship between PCB and CWB (see **Figure 1**).

## Materials and Methods

### Participants and Procedures

The participants in this study consisted of front-line coal miners and oil workers working at five large state-owned companies located in a major city in north China. The study used the survey method: We issued 570 questionnaires, and 529 were returned. Incomplete and illegible responses were removed, resulting in a final total of 484 completed surveys: a response rate of 84.9%.

Of the 484 valid respondents, 86.2% were male and 13.8% were female. The ages of participants were divided into different age groups as follows: under age 25 (15.3%), 26–35 years old (45.7%), 36–45 years old (24.8%), 46–55 years old (13.8%), and 56 years old and above (0.4%). Moreover, 3.1% of participants were educated to primary school and below, 19.4% to junior middle school, 31.4% to senior middle school, 23.3% to junior college and 22.7% to undergraduate degree level and above. With respect to job tenure, 10.5% of participants had worked for their organization less than 1 year, 13.4% had between 1 and 3 years of job tenure, 13.8% had between 3 and 5 years, 27.1% had between 5 and 10 years, and 35.1% had more than 10 years.

A cross-sectional research design was used. Data collection lasted for 1 month, from May to June 2017. Before conducting the study, we obtained permission from the ethics committee of our university, as part of a larger project on employees’ behaviors. First, we contacted the HR managers of some companies and asked them if they were willing to take part in our investigation. After obtaining permission, we met the HR managers in person to explain the purpose and benefits of the study. Finally, five companies agreed to participate in our study. Then, we distributed 114 questionnaires to each of the five companies. Each paper-and-pencil questionnaire had an attached cover letter explaining that the purpose of the survey was to examine the quality of the exchange relationship between the employees and the organization, and its implications for employees’ reactions. The letter further informed respondents that the participation was voluntary and that their privacy was strictly protected. The paper-and-pencil questionnaires were completed in a meeting room, taking a maximum of 20 min. Once completed, the questionnaires were returned to us. To symbolize our appreciation, participants were each given a notebook, which was worth 10 renminbi.

### Measures

We used a self-report questionnaire containing 46 items. Employees responded to questions regarding PCB, organizational cynicism, and work alienation on a 5-point scale, from 1 (completely disagree) to 5 (completely agree). They responded to questions on CWBs on a 7-point scale from 1 (never) to 7 (every day). Given that our samples were Chinese, the double-blind back-translation procedure (Schaffer and Riordan, 2003) was utilized to translate all items into Chinese. To facilitate understanding, each item was translated by professional translators. The internal consistency of each scale was measured using Cronbach’s alpha.

#### Psychological Contract Breach

The five-item scale developed by Robinson and Morrison (2000) was employed to assess PCB. Sample items of this scale include: “I have not received everything promised to me in exchange for my contributions” and “My organization has broken many of its promises to me even though I’ve upheld my side of the deal.” The Cronbach’s alpha for the scale for this sample was 0.80.

#### Organizational Cynicism

A 14-item scale to measure organizational cynicism was adopted from the work of Dean et al. (1998). Examples of statements were “When my organization says it’s going to do something, I wonder if it will really happen,” and “I often experience anxiety when I think about my organization.” Three dimensions of organizational cynicism were measured, i.e., organizational cynicism belief (α = 0.86), organizational cynicism affect (α = 0.73) and organizational cynicism behavior (α = 0.74). The Cronbach’s alpha for the whole scale was 0.87.

#### Work Alienation

To measure two dimensions of work alienation, we used an eight-item scale developed by Banai and Reisel (2007). Personal alienation was assessed by five items (α = 0.73) and social alienation was assessed by three items (α = 0.74). Two examples were “I would prefer to live a different life than I do” and “People are too self-centered.” The Cronbach’s alpha for the scale was 0.83.

#### Counterproductive Work Behaviors

We assessed CWBs with a 19-item measure of CWBs developed by Robinson and Bennett (1995). The scale has seven items that represent CWB-I (α = 0.77), and 12 items that represent CWB-O (α = 0.96). Examples of statements are “Made fun of someone at work,” “Dragged out work in order to get overtime.” The Cronbach’s alpha of the scale was 0.94.

### Control Variables

The participant’s gender, age, education and job tenure were controlled for. This is because, according to the previous research, these variables affect the level of employee CWB (Tepper et al., 2009; Samnani et al., 2014; Neves and Champion, 2015).

### Data Analysis

The internal consistency, descriptive statistics, and correlations among the variables were analyzed using SPSS 21.0. Our hypothetical model was examined by maximum likelihood structural equation modeling (SEM) with AMOS 22.0. According to the recommendation of Anderson and Gerbing (1988), a two-step method was utilized to examine the mediation effects. The first stage was measurement model testing. In this stage, we tested the discriminate validity of the variables by performing confirmatory factor analyses (CFAs; Cheung and Wong, 2011; Choi and Moon, 2017). The fit indices of the hypothesized factor model were compared with alternative factor models to confirm which fit the observed data better (Mathieu and Farr, 1991; Cheung and Wong, 2011). In the second stage, we compared the fit indices of the proposed model with those of alternative models to make sure which was the best model after the first stage was validated (Li et al., 2013).

The following indices were used to study the adequacy of the estimated model: χ^2^/*df*, normed fit index (NFI), goodness-of-fit index (GFI), root mean square error of approximation (RMSEA), and comparative fit index (CFI). It is acceptable for χ^2^/*df* to be between one and five (Salisbury et al., 2002). The GFI, NFI, and CFI should be over 0.90 (Salisbury et al., 2002), and the value of RMSEA should be less than 0.08 (Byrne, 2006).

## Results

### Common Method Variance

Common method variance (CMV) refers to the inflation of correlations between variables when a self-reported questionnaire is used to gather data (Podsakoff et al., 2003). This may lead to false support for the hypotheses. To test whether CMV was a problem, we employed Harman’s single-factor test. We loaded all items for each variable into a factor analysis (Podsakoff et al., 2003). The result indicated that the first factor explained 21.02% of the variance, far below 50%, demonstrating that CMV was not a problem in this research (Hair et al., 1998).

### Descriptive Statistics

The means and standard deviations of the study variables, and correlations between each of the variables are shown in **Table 1**. As we expected, PCB, organizational cynicism, work alienation and CWB were significantly correlated with each other. Among them, PCB was positively associated with CWB (*r* = 0.29, *p* < 0.001), organizational cynicism (*r* = 0.18, *p* < 0.001) and work alienation (*r* = 0.30, *p* < 0.001). Moreover, organizational cynicism was positively associated with CWB (*r* = 0.30, *p* < 0.001) and work alienation (*r* = 0.46, *p* < 0.001). Also, work alienation was positively correlated with CWB (*r* = 0.50, *p* < 0.001). Additionally, following Becker (2005) suggested that control variables should be put into the model if they correlated with the outcome variables. However, the correlations between control variables and our study variables were weak or nonsignificant; thus, the effect of control variables was not taken into consideration in our subsequent analysis. These results provided preliminary support to H1, H2, H3, H5, H6, and H8 and provided the foundation for subsequent analyses.

**Table 1:** Descriptive statistics and correlations among all variables.

Variables	*M*	*SD*	1	2	3	4	5	6	7	8
(1) Gender	0.15	0.37								
(2) Age	2.39	0.92	-0.28^∗∗∗^							
(3) Education	3.44	1.13	0.25^∗∗∗^	-0.39***						
(4) Job tenure	3.62	1.36	-0.41^∗∗∗^	0.61***	-0.36***					
(5) Psychological contract breach	3.16	0.81	0.01	0.12**	-0.07	0.05	(0.80)			
(6) Organizational cynicism	3.14	0.54	-0.01	0.06	-0.02	0.01	0.18***	(0.87)		
(7) Work alienation	3.13	0.81	-0.06	0.06	0.02	0.07	0.30***	0.46***	(0.83)	
(8) Counterproductive work behavior	4.12	1.34	-0.05	0.09	-0.05	0.04	0.29***	0.30***	0.50^∗∗∗^	(0.94)


**N* = 484. Gender: 0 = male, 1 = female; Age: 1 = under age 25, 2 = 26–35-year-old, 3 = 36–45-year-old, 4 = 46–55-year-old, 5 = 56-year-old and above; Education: 1 = primary school and below, 2 = junior middle school, 3 = senior middle school, 4 = junior college, 5 = bachelor degree and above; Job tenure: 1 = under 1year, 2 = 1–3 years, 3 = 3–5 years, 4 = 5–10 years, 5 = above 10 years Cronbach’s alpha are in parentheses on the diagonal. *^∗∗^p < 0*.01; *^∗∗∗^p < 0*.001.*

### Measurement Model Testing

Before examining the hypotheses, we performed a confirmatory factor analysis (CFA) using AMOS 22.0 to test the discriminant validity of our core variables. To minimize the magnification of measurement error for latent variables, researchers have argued that item parcels should be created to act as indicators of variables with no sub-scales (Rogers and Schmitt, 2004). This can improve the reliability and normality of measurement (Yu et al., 2016). Therefore, two-item parcels were developed for PCB. Finally, this study contained nine observed indicators and four latent factors (PCB, organizational cynicism, work alienation and CWB; see **Figure 4**). Compared to item-level data, item parcels have shown some advantages (e.g., higher communality and lower random error; Matsunaga, 2008). Using a chi-square test to compare 1-factor, 2-factor, 3-factor, and 4-factor models, we found that the 4-factor model (PCB, organizational cynicism, work alienation and CWB being independent of each other) fit the data better (χ^2^/*df* = 4.08, GFI = 0.94, NFI = 0.90, CFI = 0.91, RMSEA = 0.076; see **Table 2**).

**Table 2:** Comparison of measurement model.

Structure	χ^2^	*df*	χ^2^/*df*	GFI	NFI	CFI	RMSEA
4-factor	85.68	21	4.08	0.94	0.90	0.91	0.076
3-factor	271.67	24	11.30	0.89	0.80	0.83	0.151
2-factor	474.60	26	18.25	0.82	0.65	0.66	0.190
1-factor	535.21	27	19.82	0.80	0.60	0.61	0.201


*1-factor, PCB+OC+WA+CWB; 2-factor: OC, PCB+WA+CWB; 3-factor, PCB, OC, WA+CWB; 4-factor, PCB, OC, WA, CWB; PCB, psychological contract breach; OC, organizational cynicism; WA, work alienation; CWB, counterproductive work behavior.*

### Model Structure Testing

**FIGURE 2:**
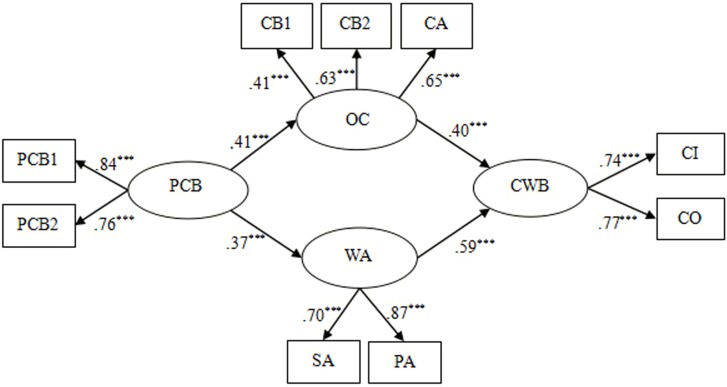
Fully mediated model (Model 1). PCB, psychological contract breach; OC, organizational cynicism; WA, work alienation; CWB, counterproductive work behaviors; PCB1 aggregates of two items and PCB2 is three items from Psychological Contract Breach Questionnaire; CB1, cynicism belief, CB2, cynicism behavior, CA, cynicism affect; CB1, CB2, and CA are three dimensions of the Organizational Cynicism Questionnaire; SA, social alienation, PA, personal alienation; SA and PA are the two dimensions of Work Alienation Questionnaire; CI, CWB-I; CO, CWB-O; CI and CO are two dimensions of the Counterproductive Work Behavior Questionnaire. ^∗∗∗^*p* < 0.001.

The present study used the maximum likelihood method in AMOS 22.0 to test the mediating effects of organizational cynicism and work alienation. We established several alternative models to test the mediating effects. First, we built Model 1 (a fully mediated model), in which PCB only indirectly related to CWBs through organizational cynicism and work alienation (see **Figure 2**). The result indicated that Model 1 (χ^2^*/df* = 3.94, GFI = 0.96, NFI = 0.95, CFI = 0.96, RMSEA = 0.078) showed a good fit to the data (see **Table 3**).

**Table 3:** Comparison of the structural models.

Model	χ^2^	*df*	χ^2^/*df*	GFI	NFI	CFI	RMSEA
M1 (fully mediated model)	90.62	23	3.94	0.96	0.95	0.96	0.078
M2 (partially mediated model)	170.72	22	7.76	0.95	0.91	0.92	0.118
M3 (The final model)	38.34	22	1.74	0.98	0.97	0.98	0.039


**FIGURE 3:**
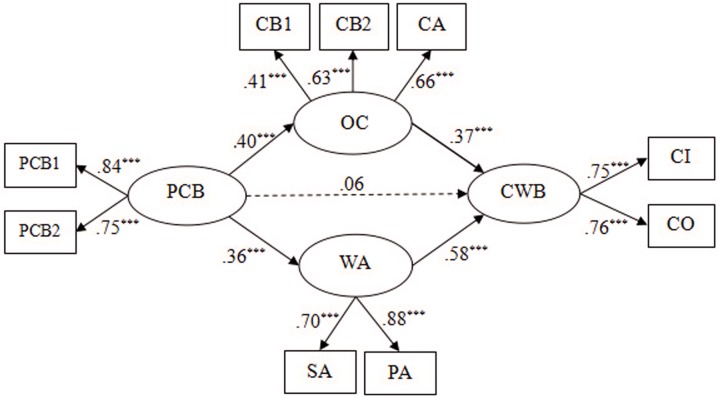
Partially mediated model (Model 2). PCB, psychological contract breach; OC, organizational cynicism; WA, work alienation; CWB, counterproductive work behaviors; PCB1 aggregates of two items and PCB2 is three items from Psychological Contract Breach Questionnaire; CB1, cynicism belief, CB2, cynicism behavior, CA, cynicism affect; CB1, CB2, and CA are three dimensions of the Organizational Cynicism Questionnaire; SA, social alienation, PA, personal alienation; SA and PA are the two dimensions of Work Alienation Questionnaire; CI, CWB-I; CO, CWB-O; CI and CO are two dimensions of the Counterproductive Work Behavior Questionnaire. ^∗∗∗^*p* < 0.001.

Second, we added a direct path from PCB to CWBs based on Model 1, and thus Model 2 (partially mediated model) was set up (see **Figure 3**). The results revealed that Model 2 (χ^2^/*df* = 7.76, GFI = 0.95, NFI = 0.91, CFI = 0.92, RMSEA = 0.118) did not fit the data well. We also found that the direct effect from PCB to CWB was not significant (β = 0.06, *p* > 0.05). Moreover, through the comparison between Model 1 and Model 2, we found the chi-square difference reached significance, Δχ^2^_(1)_ = 80.1, *p* < 0.001, which revealed Model 1 as superior to Model 2 (see **Table 3**)

**FIGURE 4:**
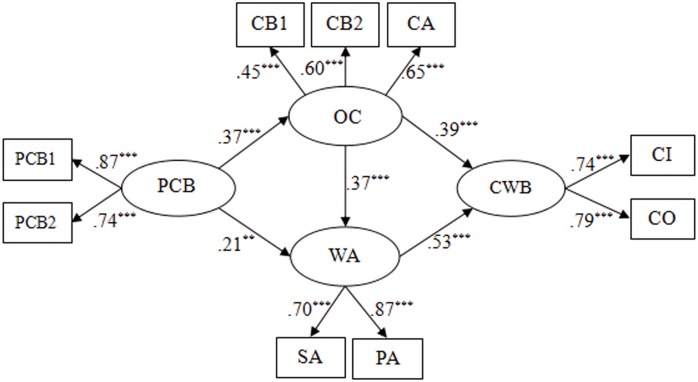
The ultimate mediation model (Model 3). PCB, psychological contract breach; OC, organizational cynicism; WA, work alienation; CWB, counterproductive work behaviors; PCB1 aggregates of two items and PCB2 is three items from Psychological Contract Breach Questionnaire; CB1, cynicism belief, CB2, cynicism behavior, CA, cynicism affect; CB1, CB2, and CA are three dimensions of the Organizational Cynicism Questionnaire; SA, social alienation, PA, personal alienation; SA and PA are the two dimensions of Work Alienation Questionnaire; CI, CWB-I; CO, CWB-O; CI and CO are two dimensions of the Counterproductive Work Behavior Questionnaire. ^∗∗^*p* < 0.01; ^∗∗∗^*p* < 0.001.

Then, to find the most satisfactory model, we developed another alternative model (Model 3), where we added a path from organizational cynicism to work alienation based on Model 1 (see **Figure 4**). The result suggested Model 3 (χ^2^/*df* = 1.74, GFI = 0.98, NFI = 0.97, CFI = 0.98, RMSEA = 0.039) also fit the data well. Moreover, each latent factor was well represented by its indicators, because factor loadings on these ranged from 0.45 to 0.87 (*p* < 0.001). By comparing the Model 1 and Model 3, we found that the chi-square difference reached significance, Δχ^2^_(1)_ = 52.28, *p* < 0.001, which revealed Model 3 as superior to Model 1 (see **Table 3**). Thus, Model 3 was selected as the ultimate structural model.

In accordance with the recommendation of Preacher and Hayes (2008), the bootstrapping method was utilized to examine the mediation effects shown in Model 3. Bootstrapping is the ideal way to test the indirect effects because it avoids non-normal sampling distributions (Zhang et al., 2015). If zero was not included in the 95% confidence interval, then the indirect effects reached a significant level. As shown in **Table 4** and **Figure 4**, the results suggested that our hypotheses were all verified. First, the total effect of PCB on CWB was notable (β = 0.33, *p* < 0.001), supporting H1. Second, PCB had a positive effect on organizational cynicism (β = 0.37, *p* < 0.001), and thus H2 was supported. Third, the direct effect of organizational cynicism on CWB was significant (β = 0.39, *p* < 0.001), supporting H3. Fourth, the indirect effect of PCB on CWB via organizational cynicism was significant (β = 0.143, *p* < 0.001), supporting H4. Fifth, the direct effect of PCB on work alienation was significant (β = 0.21, *p* < 0.01), confirming H5. Sixth, the path coefficient between work alienation and CWB was significant (β = 0.53, *p* < 0.001), supporting H6. Seventh, the indirect effect of PCB on CWB via work alienation was significant (β = 0.110, *p* < 0.01). Eighth, organizational cynicism had a significant positive effect on work alienation (β = 0.37, *p* < 0.001), supporting H8. Furthermore, we also demonstrated that the link between PCB and CWB was sequentially mediated by organizational cynicism and work alienation (β = 0.071, *p* < 0.01).

**Table 4:** Direct and indirect effects and95% confidence intervals in ultimate model 3.

Model pathways	Estimated effect	95% CI	
		
		Lower bounds	Upper bounds
**Total Effect**			
PCB → CWB	0.33***	0.219	0.416
**Direct Effects**			
PCB → OC	0.37***	0.186	0.522
PCB → WA	0.21**	0.080	0.339
OC → CWB	0.39***	0.218	0.553
OC → WA	0.37***	0.223	0.503
WA → CWB	0.53***	0.400	0.651
**Indirect Effects**			
PCB → OC → CWB	0.143***	0.049	0.238
PCB → WA → CWB	0.110**	0.011	0.210
CB → OC → WA → CWB	0.071**	0.024	0.119


*PCB, psychological contract breach; OC, organizational cynicism; WA, work alienation; CWB, counterproductive work behaviors. ^∗∗^*p* < 0.01, ^∗∗∗^*p* < 0.001.*

## Discussion

Counterproductive work behaviors bring losses to the organization directly or indirectly and have gained much research attention (Chernyak-Hai and Tziner, 2014). However, there was little previous research investigating the formative mechanisms of CWB from the perspective of PCB. In this study, through analysis and comparison between our hypothetical model and alternative models, we found that the relationship of PCB with CWB was mediated by work alienation and organizational cynicism respectively. Moreover, organizational cynicism and work alienation sequentially mediated the relationship between PCB and CWB.

### Theoretical Implications

This research has some vital theoretical implications. By drawing on social exchange theory, we provided strong empirical evidence to aid our understanding of the underlying mechanism of the PCB-CWB relationship. When employees perceive that the exchange relationship with their organization is disrupted, they exhibit more negative outcomes (Joe et al., 2011). In this light, the findings of the present study suggested that PCB was significantly positively related to CWB, which is in accordance with previous research (e.g., Özdemir and Demircioglu, 2015; Griep and Vantilborgh, 2018). Furthermore, the relation between PCB and CWB was mediated by organizational cynicism and work alienation, which is consistent with the view of Ajzen (1989) that attitude acts as a bridge between perception and behavior. Thus, by establishing the causal linkage among perception, attitude, and behaviors, the present study confirms social exchange theory, which postulates that perceptions cause attitudes, which then cause behaviors (Blau, 1964). Specifically, whenever employees perceive that they do not obtain the reciprocal return from the organization, they respond with negative attitudes (e.g., organizational cynicism and work alienation), which in turn causes them to engage in CWBs in return to restore the reciprocity.

Furthermore, researchers have previously tended to prefer to treat positive attitudes, like trust (Liu et al., 2013; Guo, 2017) and job satisfaction (Turnley and Feldman, 2000; Balogun et al., 2017) as mediators in the link between PCB and behavioral outcomes, which could lead to a one-sided understanding of the role of attitudes. In this study, we took negative attitudes (i.e., organizational cynicism and work alienation) as mediators. The results showed that PCB not only directly affects organizational cynicism and work alienation but also affects CWB via the mediating effects of organizational cynicism and work alienation. Therefore, our work revealed that negative attitudes also play a significant role in the relationship between PCB and CWB. To our knowledge, this is the first study to bring organizational cynicism and work alienation as mediators into the link between PCB and CWB. Hence, this study helps us to understand the role of attitudes.

### Practical Implications

The outcomes of the study have practical implications for the energy industry in China, in terms of controlling CWB. First, given that PCB can trigger a series of negative work-related thoughts and behaviors (organizational cynicism, work alienation, and CWB), the industry needs to prevent CWB from happening. To achieve this, organizations should provide realistic promises during recruitment, socialization, and routine work interactions and try to fulfill the reasonable psychological contract of employees. However, most organizations are under intense pressure to frequently change their organization. It would seem to be unrealistic to fulfill each promise made to a job incumbent (Bordia et al., 2008). In this case, organizations can perform some interventions and remedies. For example, it has been shown that resource-based intervention programs that build the psychological capital of employees help to improve employees’ work engagement and to build strong relationships between employees and the organization (Costantini et al., 2017). In addition, the organization can obtain the employees’ understanding by explaining the reason for the breach and compensating the employees in other ways (Morrison and Robinson, 1997).

Second, because organizational cynicism is positively correlated with work alienation and CWB, organizations should strive to eliminate the effect of organizational cynicism. Organizations can increase trustworthiness in numerous ways. A supportive and fair culture should be established to reduce the frequency of organizational cynicism. In addition, open and honest communication is needed between employees and the organization. Communication not only serves to direct work and state policies, and to provide feedback (Katz and Kahn, 1966) but also conveys to the employees that the organization values and cares for them (Neves and Eisenberger, 2012). For the purpose of communication, organizations can hold regular meetings in which employees are encouraged to express their views and make reasonable demands openly in front of the management. If some employees dislike expressing themselves face to face, the organization can build a communication platform in which some work-related information could be shared, encouraging employees to make rational proposals and complain online. In these ways, organizational cynicism can be avoided and a harmonious exchange relationship can be established.

Finally, to reduce the influence of work alienation, an organization should create more chances for employees to participate in psychological counseling and consultation. This is an effective way for employees to reduce work alienation by building a strong relationship with their colleagues (Li and Sun, 2015). In accordance with the view of Sulu et al. (2010), empowerment serves a key role in reducing the sense of powerlessness, which is one of the important forms of work alienation. Thus, organizations can allow employees to participate in the decision-making process and give them certain powers to control their own work. Besides, extending and enriching the work and decreasing work pressure are useful to improve enthusiasm for work and to remove work alienation.

Participants in this study consisted of employees working in energy companies, and the findings suggest that PCB has significant effects on attitudes (i.e., organizational cynicism and work alienation) and behavior (i.e., CWB). In the past, many studies on PCB have used medical staff (Ahmed et al., 2013; Rodwell and Gulyas, 2013; Trybou and Gemmel, 2016) or bankers (Nwankwo et al., 2013; Kagaari, 2014) as the participants. However, the employees working at energy companies (e.g., coal miners and oil workers) were ignored in prior research. Due to the poor salary structure and career opportunities, employees working at energy companies are not satisfied. Therefore, it is important to test the effects of their PCB on attitudes and behavior. This particular study may help researchers pay more attention to employees working at energy companies. It will also help researchers who are undertaking related research applications in different industries.

### Limitations and Future Research

This study inevitably had some limitations. First, the nature of our study design was cross-sectional. Thus, we cannot make claims about causality. Some alternative explanations may not be excluded. For example, in our research, we assumed that employees with high levels of organizational cynicism or work alienation were more prone to engaging in CWB. Another possibility is that due to their CWB, employees were punished by their organization, leading to organizational cynicism or work alienation. Therefore, to produce exact conclusions about causality, longitudinal and experimental studies should be adopted in future.

Second, we assessed our variables through a self-report questionnaire. Due to social desirability response bias, participants tend to conceal their real responses (Podsakoff and Organ, 1986). Thus, the real frequency of CWB was likely under-reported by participants to avoid being identified and punished by their organization. However, Spector and Fox (2002) argued that CWB measures are often limited to self-reports by necessity as these types of behaviors are “carefully hidden.” Accordingly, in future research, we recommend that researchers should examine whether differences exist between other-reports (leader-assessment, coworker-assessment, and partner-assessment) and self-reports.

Finally, the generalizability of the present study may be a problem. Our participants were all Chinese, from five energy companies, and these may not even accurately represent all the energy companies in China. Moreover, different organizations may have different HR practices and organizational cultures, which could produce different conclusions. Thus, in accordance with the recommendation of Marôco (2010), researchers on future should test our model with distinct samples and in more diverse industries to allow greater generalization.

## Ethics Statement

This study was carried out in accordance with the recommendations of ethics committee of China University of Mining and Technology with written informed consent from all subjects. All subjects gave written informed consent in accordance with the Declaration of Helsinki. The protocol was approved by the ethics committee of China University of Mining and Technology.

## Author Contributions

SL collected and analyzed data for the study. YC designed and drafted the work.

## Conflict of Interest Statement

The authors declare that the research was conducted in the absence of any commercial or financial relationships that could be construed as a potential conflict of interest. The reviewer AC and handling Editor declared their shared affiliation.
